# The impact of oxidative stress and the NRF2-KEAP1-ARE signaling pathway on anticancer drug resistance

**DOI:** 10.32604/or.2025.065755

**Published:** 2025-07-18

**Authors:** FLáVIA ALVES VERZA, GUILHERME CARVALHO DA SILVA, FELIPE GARCIA NISHIMURA

**Affiliations:** 1 Department of Pharmacology, Ribeirao Preto Medical School, University of São Paulo, Ribeirão Preto-SP, 14040-900, Brazil; 2 Department of Clinical Analyses, Toxicology and Food Sciences, School of Pharmaceutical Sciences of Ribeirão Preto, University of São Paulo, Ribeirão Preto-SP, 14040-903, Brazil

**Keywords:** Reactive oxygen species, Antioxidant response, Tumor Protein 53, Therapy resistance

## Abstract

Cancer remains a major global health burden, with rising incidence and mortality linked to aging populations and increased exposure to genotoxic agents. Oxidative stress plays a critical role in cancer development, progression, and resistance to therapy. The nuclear factor erythroid 2-related factor 2 (NRF2)-Kelch-like ECH-associated protein 1 (KEAP1)-antioxidant response element (ARE) signaling pathway is central to maintaining redox balance by regulating the expression of antioxidant and detoxification genes. Under physiological conditions, this pathway protects cells from oxidative damage, however, sustained activation of NRF2 in cancer, often due to mutations in KEAP1, supports tumor cell survival, drug resistance, and metabolic reprogramming. Recent studies demonstrate that NRF2 enhances glutathione (GSH) synthesis, induces detoxifying enzymes, and upregulates drug efflux transporters, collectively contributing to resistance against chemotherapy and targeted therapies. The inhibition of NRF2 using small molecules or dietary phytochemicals has shown promise in restoring drug sensitivity in preclinical cancer models. This review highlights the dual role of NRF2 in redox regulation and cancer therapy, emphasizing its potential as a therapeutic target. While targeting NRF2 offers a novel approach to overcoming treatment resistance, further research is needed to enhance specificity and facilitate clinical translation.

## Introduction

Malignant diseases can be regarded as the first and foremost public health-care challenge in public health worldwide. It accounts for nearly one in six deaths (16.8%) all over the world and represents 22.8% of deaths from noncommunicable diseases (NCDs) [1]. Additionally, cancer is responsible for 30.3% of premature deaths from NCDs among individuals aged 30 to 69, ranking among the top three causes of death in this age group in 177 out of 183 countries [2]. The rising incidence of cancer and cancer-related deaths each year is driven by a combination of interrelated factors, including population growth, aging demographics, and changes in lifestyle and environmental exposures [3]. Both external (environmental and lifestyle-related) and internal (genetic and physiological) factors contribute to sustained exposure to genotoxic agents, which increases the risk of developing cancer [4]. Additionally, demographic transitions and inherited genetic predispositions further compound this risk by extending the duration of exposure to carcinogenic influences [5]. Environmental, exogenous, and endogenous factors also play a crucial role in influencing cancer incidence. Exposure to environmental carcinogens such as pollutants, radiation, and industrial chemicals can induce DNA damage and genetic mutations, ultimately contributing to the initiation and progression of carcinogenesis.

Cancer is a complex disease characterized by uncontrolled cell growth and the ability to evade regulatory mechanisms that maintain cellular homeostasis. Each different type of cancer has unique causes and symptoms [6]. Among the key biological processes influencing tumor development and progression are oxidative stress, inflammation, and apoptosis, which interact in a dynamic and often dysregulated manner in cancer cells. Oxidative stress, caused by an imbalance between reactive oxygen species (ROS) production and antioxidant defenses, contributes to DNA damage, genetic mutations, and the activation of oncogenic signaling pathways [7,8]. Chronic inflammation further promotes tumorigenesis by creating a pro-tumor microenvironment rich in cytokines, growth factors, and ROS, which sustain cancer cell proliferation, survival, and metastasis [9]. Apoptosis, a programmed cell death mechanism essential for eliminating damaged or abnormal cells, is frequently disrupted in cancer, allowing malignant cells to evade death and resist therapy [10]. Another challenge in cancer treatment is overcoming therapy resistance, which arises from a variety of factors, including tumor size, heterogeneity, the tumor microenvironment, and the immune system. On top of that, the presence of undruggable cancer drivers and the treatment pressures themselves add more layers of complexity [11]. In cancers like prostate cancer, the development of castration-resistant prostate cancer (CRPC) within a few years of androgen deprivation therapy is common, leading to a poor prognosis and reduced survival rates [12]. Similarly, in multiple myeloma, drug resistance limits effective treatment options for patients not eligible for stem cell transplants, making it a difficult cancer to treat [13]. Drug resistance is responsible for a large proportion of treatment failures and patient deaths, with studies showing it contributes to over 90% of cancer-related deaths in some cases [14]. Beyond the clinical impact, drug resistance also imposes a heavy economic burden on healthcare systems, as patients require alternative therapies, extended hospital stays and face a financial strain due to prolonged illness and lost productivity [15]. The need to address these issues calls for a multifaceted approach, including early detection, personalized therapies, and the development of novel treatments to overcome resistance mechanisms, ultimately improving patient outcomes and reducing the economic strain.

Understanding the intricate relationship between these processes provides critical insights into cancer pathophysiology and opens new avenues for therapeutic interventions targeting oxidative stress, inflammatory pathways, and apoptotic regulation. In this review, we aim to provide a comprehensive overview of the role of oxidative stress in cancer development and progression, with a particular focus on the NRF2–KEAP1–ARE signaling pathway. We explore how this pathway contributes to both tumor suppression and tumor promotion, depending on the cellular context, and how its modulation may offer novel therapeutic opportunities. Additionally, we discuss current and emerging strategies for targeting this pathway in cancer therapy, including pharmacological activators and inhibitors, and evaluate their potential to overcome therapeutic resistance and improve clinical outcomes.

## The DNA Damage Response

**Figure 1:**
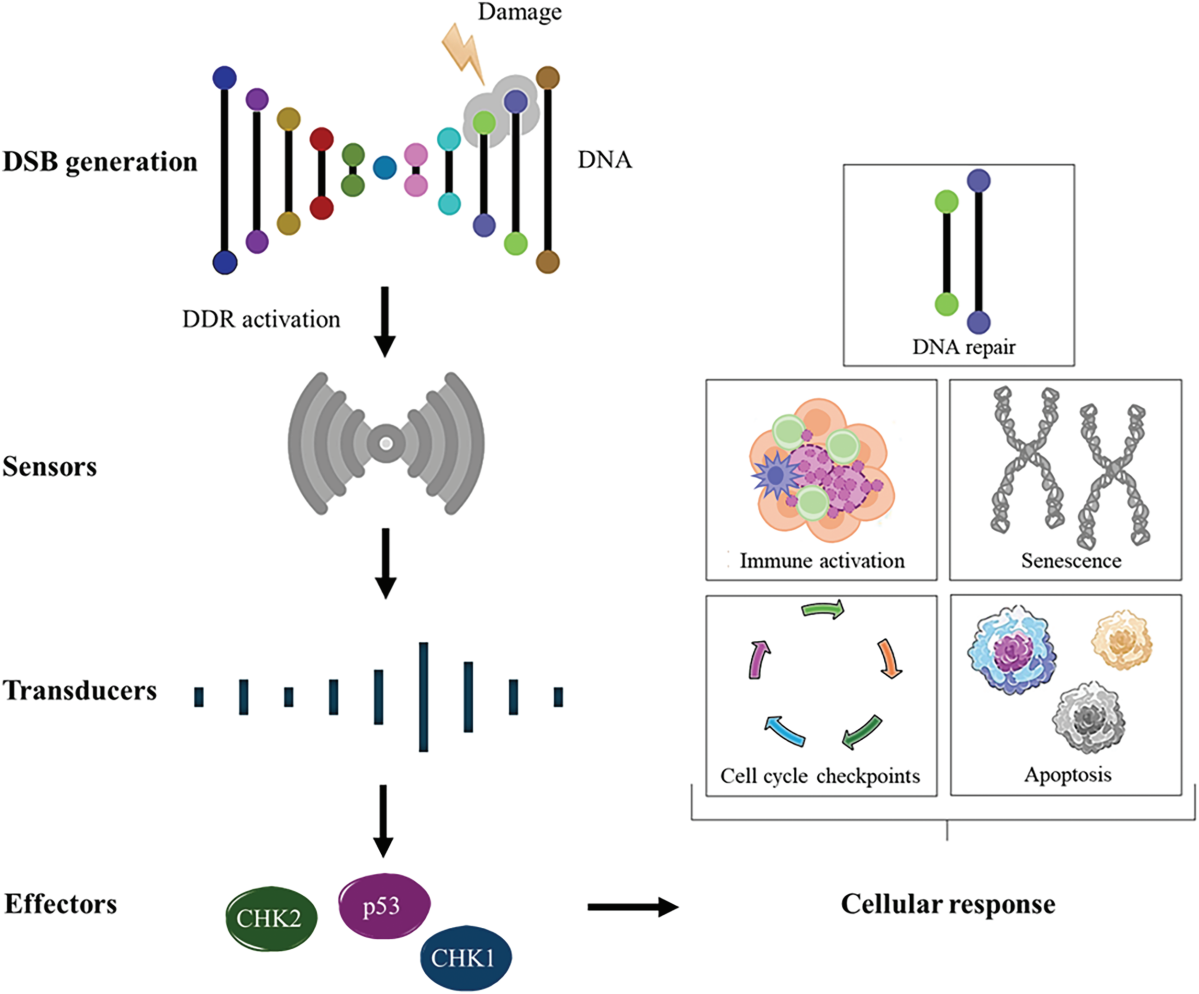
An overview of DDR activation and eventual outcome. DSBs are recognized by sensor proteins that recruit and activate signal transducers at the site of damage, leading to the activation of key effector proteins such as CHK1, CHK2, and p53, which coordinate the cellular DNA damage response. Notes: DSB, double-strand break; CHK, checkpoint kinases; DDR, DNA damage response. Adapted from [23,24].

The DNA molecule is the primary target of many anticancer drugs used clinically in treatment therapies. The impact of these compounds on the stability of the cellular genome reflects their ability to interact with cellular DNA and the cells’ capacity to neutralize or attenuate the effects of this interaction. The influence of anticancer drugs on DNA damage is critical to their mechanism of action, but it also has implications for both tumor cells and normal cells [16]. DNA damage refers to alterations in the DNA molecule that can affect the interpretation and transmission of genetic information at that site. DNA can be damaged by various exogenous and endogenous agents, including chemicals, radiation, free radicals, and topological changes, each causing distinct forms of damage [17]. Potential sources of DNA damage include endogenous factors, such as those generated by metabolic activities like ROS and DNA replication, as well as exogenous factors, such as environmental agents (ultraviolet [UV] rays and ionizing radiation) or chemotherapeutic agents [18]. These DNA damages create structural alterations that can impair gene transcription and DNA replication, compromising vital cellular functions [19]. To neutralize the constant occurrence of DNA lesions, cells have developed complex repair systems responsible for maintaining the integrity of genetic material. Depending on the nature of DNA damage, specific pathways are activated to identify the damaged regions and initiate the DNA damage response (DDR) [20]. The DDR is organized into distinct, yet functionally interconnected, pathways, each primarily defined by the specific type of DNA damage it addresses. DDR pathways generally follow a well-coordinated sequence of events, the detection of DNA damage, the accumulation of repair factors at the damage site and the repair of the lesion [21]. One particularly cytotoxic lesion is the double-strand break (DSB), which can lead to mutagenic effects due to chromosomal rearrangements or loss of genetic information resulting from faulty DNA repair [22]. The response to this damage involves damage recognition by “sensors,” activation of cell cycle checkpoints and arrest mediated by “transducers,” and, ultimately, repair and apoptosis through “effectors” that modulate the cellular outcomes, ensuring genomic stability or triggering cell death when necessary [20] (Fig. 1).

Other pathways that may be activated include base excision repair (BER), which removes lesions caused by base oxidation [25]; nucleotide excision repair (NER), which removes lesions that distort the DNA double helix [26]; mismatch repair (MMR), which corrects incorrectly inserted nucleotides during DNA replication [27]; homologous recombination repair (HRR) and non-homologous end joining (NHEJ), two pathways involved in repairing double-strand breaks in DNA [28]. These DDR pathways play a crucial role in safeguarding genomic stability, a key factor in cancer development. Compared to normal tissues, precancerous and tumor cells experience heightened DNA damage and replication stress, further contributing to genomic instability. The regulation of these pathways is complex, involving various transcription factors that directly and indirectly control DNA repair genes, ensuring the proper function of the DNA repair machinery.

## Role of ROS in Signaling Pathways Activated in Human Malignancies

ROS are well-known as metabolites with strong oxidative capacity and are involved in intracellular signal transduction. These molecules, which act as oxygen carriers, have reactive properties and include radicals such as O_2_^−^ (superoxide), HO• (hydroxyl), and non-radicals like H_2_O_2_ (hydrogen peroxide) [29]. Recently, ROS has been studied as an important signaling molecules that play a significant role in the progression of various pathological conditions, such as diabetes [30], cardiovascular diseases [31], neurodegenerative diseases [32], and cancer [33]. Low to moderate levels of ROS are crucial for regulating normal biological functions, such as cell survival, growth, senescence, and aging [34]. However, an imbalance in the redox system in favor of oxidants can lead to oxidative stress, a condition characterized by the overproduction of ROS and/or a decrease in antioxidant capacity. Interestingly, ROS also influences the tumor microenvironment and is known to initiate cancer angiogenesis, metastasis, and survival through the activation of various signaling pathways, including nuclear factor kappa-B (NF-κB), and phosphatidylinositol 3-kinase (PI3K)/protein kinase B (Akt) [35]. ROS are chemically reactive molecules that can cause damage to DNA, proteins, and lipids, leading to genomic instability, a hallmark of cancer. This instability arises from the accumulation of DNA mutations and chromosomal aberrations, which can promote tumorigenesis [36]. The imbalance between ROS production and the antioxidant defense system results in oxidative stress, which further exacerbates DNA damage and contributes to cancer progression [37]. Therefore, it is essential to regulate ROS inducers and their elimination pathways effectively. Although ROS are natural byproducts of metabolic processes, they can interact with proteins, lipids, and epigenetic modifications that influence gene expression, potentially inducing direct damage to these molecules, which can lead to mutations in normal cells, resulting in genomic instability and cancer progression [38]. Altered cells exhibit a high metabolic rate due to their rapid division, which can increase DNA oxidation and induce the excessive generation of oxidative stress markers, making ROS a key regulatory mechanism for tumor cells [39]. During tumor progression, ROS can stimulate the growth of mutant cells by modulating genes responsible for cell proliferation or death. Redox homeostasis is controlled by an extensive machinery of enzymes and non-enzymatic compounds. Some of the main transcription factors involved in redox regulation include nuclear respiratory factor 1 (NRF1) [40], nuclear factor erythroid 2-related Factor 2 (NRF2) [41], and Tumor Protein 53 (p53) [42]. The disruption of redox balance due to excessive ROS production leads to oxidative stress, which can result in mutations that activate oncogenes or inactivate tumor suppressor genes. ROS-induced DNA damage includes base modifications, strand breaks, and cross-linking, which can lead to mutations if not properly repaired [43]. Although ROS encompasses various molecular species, each contributes differently to malignant processes, and our understanding of their roles in cancer remains incomplete. Further research is needed to clarify the biological mechanisms associated with ROS and their impact on tumor progression.

## Oxidative Stress Modulation in Cancer Treatment

Targeting oxidative stress in cancer therapy presents both promising opportunities and significant challenges. ROS play a dual role in cancer biology, acting as both promoters and suppressors of tumor growth. Cancer cells typically exhibit elevated ROS levels due to their altered metabolism, which can drive oncogenic signaling and genomic instability. However, this increased ROS burden also makes cancer cells more susceptible to oxidative stress-induced cell death, presenting a potential therapeutic target [44]. The therapeutic interventions can be broadly divided into increasing ROS levels beyond the threshold of tolerance in cancer cells to induce cytotoxicity and enhancing antioxidant defenses to protect normal cells or prevent cancer initiation [37]. Despite its therapeutic potential, targeting oxidative stress comes with significant challenges. The redox paradox highlights the difficulty in determining whether to inhibit or enhance oxidative stress, as both approaches can be beneficial or detrimental depending on the context. Selectivity remains a significant concern, as systemic modulation of redox balance can affect both malignant and healthy tissues [45]. Moreover, many tumors develop resistance through adaptive responses involving the upregulation of antioxidant pathways, particularly through NRF2 overactivation [46]. The timing, dosing, and combination strategies of ROS-modulating therapies require careful optimization to avoid resistance and collateral damage [47]. However, this therapeutic approach faces several important obstacles. A key concern is the risk of harming healthy tissues, since unchecked oxidative stress can damage normal cells and cause toxicity [48]. In addition, cancer cells may adapt by boosting their antioxidant defenses, making them more resistant to oxidative stress and potentially reducing the effectiveness of pro-oxidant treatments [49]. Another important hurdle is the complex and dual role of ROS within the tumor microenvironment. While ROS can help kill cancer cells, they can also promote immune suppression, which interferes with the body’s natural ability to fight tumors [50]. For instance, ROS can mediate the immunosuppressive effects of myeloid-derived suppressor cells, which can hinder the activity of antitumor immune cells like T cells and natural killer cells. This necessitates a careful balance in modulating ROS levels to enhance therapeutic efficacy without compromising immune function, too much can harm the immune system, and too little might not be effective against the tumor [47]. Future research in this field is focused on developing combination therapies that pair ROS modulation with other treatments, like immunotherapy, to overcome resistance and improve outcomes. By combining these strategies, treatments could become more effective at targeting tumors. Additionally, identifying biomarkers could help doctors personalize therapies, allowing for more precise treatments tailored to each patient and minimizing side effects [51]. Overall, while using oxidative stress to treat cancer holds great promise, it requires a careful, balanced approach to tackle the challenges and achieve the best possible results for patients.

## NRF2-KEAP1-ARE Pathway

**Figure 2:**
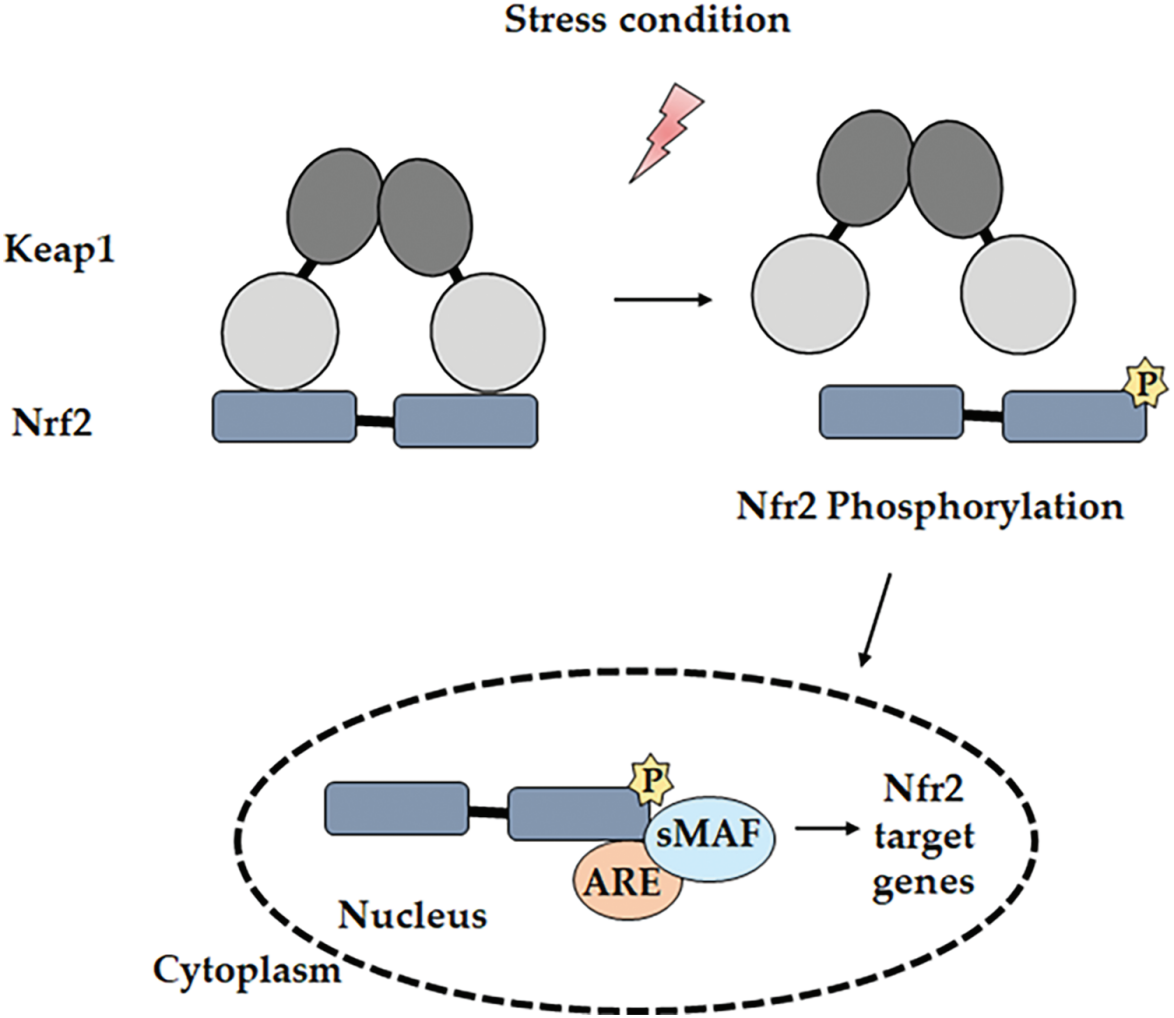
Mechanism of the NRF2 pathway. Under oxidative stress conditions, NRF2 dissociates from KEAP1 through its phosphorylation by various kinases. NRF2 then translocates into the nucleus and binds to the ARE region on the promoter of the target gene. Notes: P, phosphorylation. Adapted from [37].

One of the most critical molecular pathways involved in regulating cellular oxidative stress and promoting cancer cell survival is the NRF2-KEAP1 pathway. This pathway is a key regulator of oxidative stress, primarily controlling the expression of antioxidant and cytoprotective genes [52]. Its activity is influenced by various factors, including activating stimuli, interactions with other transcription factors, regulatory proteins, and crosstalk with multiple signaling pathways, making it a highly complex and dynamic system [53]. The gene expression of antioxidants that eliminate ROS is promoted by NRF2 [54], a member of the basic leucine zipper (bZIP) transcription factor subfamily belonging to the Cap ‘N’ Collar (CNC) family. NRF2 was identified through the analysis of its coding region, where its DNA-binding domain showed significant homology with the Nuclear Factor, Erythroid 2 (NF-E2) in the β-globin gene cluster [55]. NRF2 is a modular protein containing seven functional domains, known as NRF2-ECH homology (Neh) domains—Neh1 to Neh7—suitable for ubiquitin conjugation. The Neh1 domain contains the bZIP region necessary for heterodimerization with members of the small musculoaponeurotic fibrosarcoma (sMaf) family and for DNA binding [56]. The Neh2 domain contains two key motifs, DLG and ETGE, which are critical for binding to KEAP1. This interaction facilitates the ubiquitination and subsequent proteasomal degradation of NRF2, thereby keeping its levels low under homeostatic conditions [57,58]. The ETGE motif has a high affinity for KEAP1, while the DLG motif provides a secondary binding site, allowing for a dual interaction that stabilizes the NRF2-KEAP1 complex. This complex formation is necessary for the recruitment of the E3 ubiquitin ligase complex, which tags NRF2 for degradation [59,60]. Under basal conditions, NRF2 remains in the cytoplasm, associated with its regulatory protein, KEAP1, maintaining low levels to prevent unnecessary expression of its target genes. However, when the cell undergoes stress stimuli, such as increased ROS, specific reactive cysteine residues in KEAP1 become oxidized, leading to conformational changes and the subsequent release of NRF2 [61]. Free NRF2 molecules translocate to the nucleus and associate with sMaf proteins. This newly formed heterodimer binds to the antioxidant response element (ARE) or electrophile response element (EpRE) located in the promoter region of target genes [62]. The NRF2/sMaf complex, upon binding to ARE or EpRE, initiates transcription, triggering an antioxidant response [63] (Fig. 2).

Recent insights suggest that NRF2 also participates in a variety of DDR pathways and plays important roles in maintaining genome stability by modulating the expression of genes involved in this process and maintaining redox homeostasis, which is essential for the proper functioning of DDR mechanisms [64]. This interaction is particularly relevant in cancer, however, NRF2 sustained activation boosts the expression of genes that help cancer cells survive by strengthening their antioxidant defenses, altering their metabolism, and promoting autophagy, all of which enable them to cope with oxidative stress and continue proliferating [65]. Furthermore, loss of KEAP1 function or mutations that lead to persistent NRF2 activation can deregulate DDR, promoting the survival of cells with damaged DNA, contributing to tumorigenesis and therapy resistance [66].

**Figure 3:**
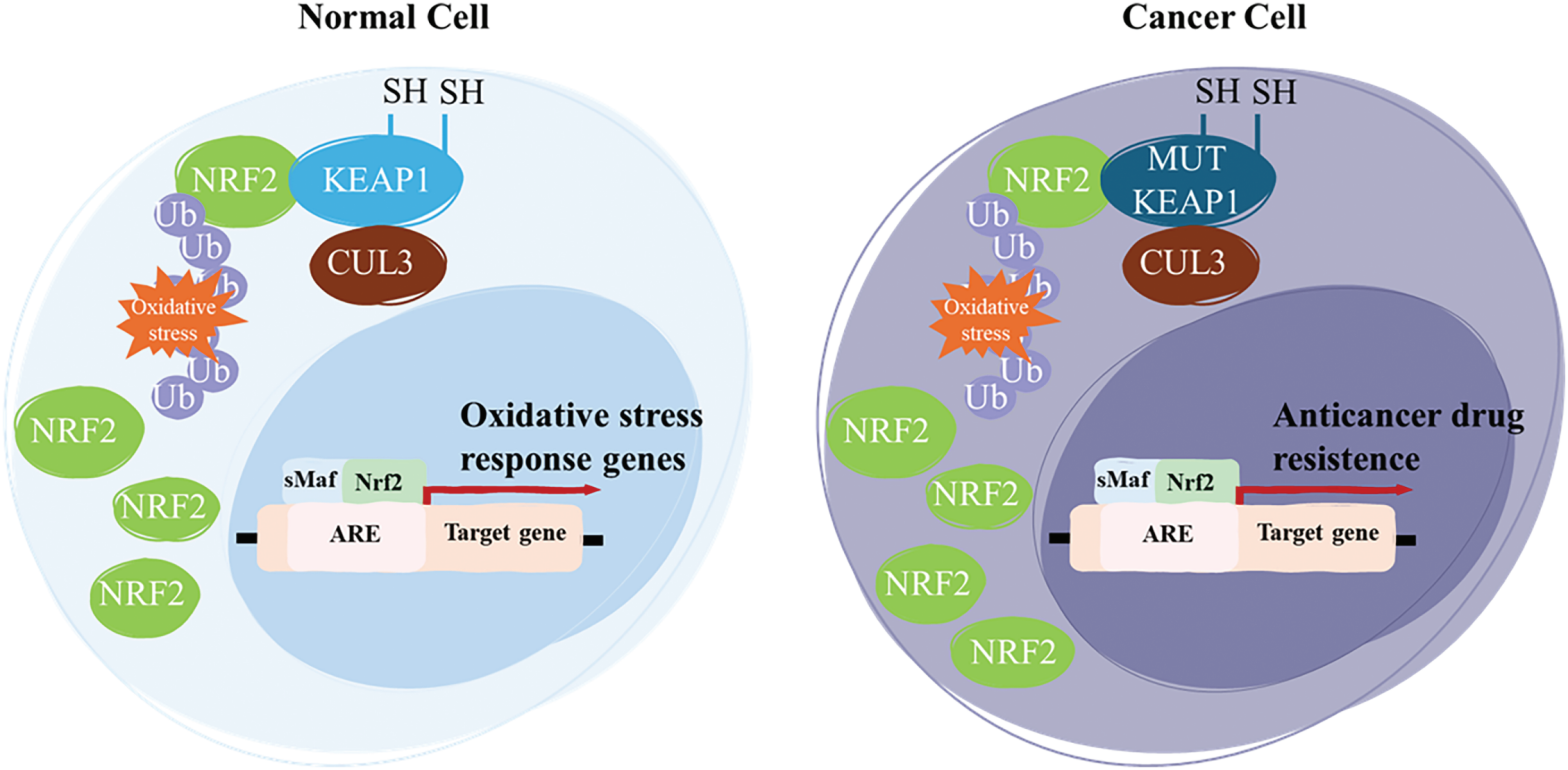
NRF2 is a key transcriptional regulator of the cellular oxidative stress response. In normal cells, NRF2 levels are transiently elevated in response to oxidative stress, allowing for the activation of cytoprotective genes. However, in cancer, aberrant activation of NRF2 is commonly driven by loss-of-function mutations in KEAP1. These mutations impair KEAP1’s ability to bind NRF2, leading to its stabilization and persistent activation. As a result, sustained NRF2 activity enhances the expression of genes involved in detoxification, survival, and ultimately, anticancer drug resistance. Notes: Ub, ubiquination; MUT, mutant; SH, thiol groups. Adapted from [77].

KEAP1 is a 69 kDa protein that contains 27 cysteine residues and belongs to the BTB (bric-a-brac, tramtrack, and broad complex)-Kelch protein family. The BTB domain comprises approximately 50 members classified as Kelch-like (KLHL1-42, with KEAP1 classified as KLHL19) or Kelch, and it possesses the BTB 1-14 domain (KBTBD1-14), which often functions as a multi-subunit complex [67,68]. The BTB domain of KEAP1 is in its N-terminal region, while the IVR and Kelch domains are in its C-terminal region [69]. The Kelch domain consists of six repeats that fold into a six-bladed β-propeller structure and is responsible for binding the BTB domain to the DLG and ETGE amino acid sequences of NRF2. Like other members of the BTB-Kelch protein family, KEAP1 associates with Cullin 3 (CUL3) and Ring-box 1 (RBX1) proteins to form a Cullin-RING E3 ligase complex that targets substrates for ubiquitination. KEAP1 binds to Cul3 through its BTB domain and the 3-box of the IVR domain, which acts as a sensor, containing most of the reactive cysteines [70]. Overall, the NRF2-KEAP1 pathway’s interaction with the DDR underscores its dual role in both protecting against and contributing to disease processes, depending on the context and extent of its activation. In cancer prevention, NRF2 activation plays a crucial role in protecting healthy cells from oxidative stress and cancer development. When activated, NRF2 boosts the expression of genes involved in protecting cells from damage. These genes help to neutralize ROS and other toxins that can cause oxidative damage [71]. Research into phytochemicals and food components that offer chemopreventive potential by enhancing NRF2-driven antioxidants and detoxifying proteins, which help eliminate electrophile-induced carcinogenesis, is a rapidly growing area in cancer prevention studies [72]. Dietary phytochemicals can help prevent diseases by reducing oxidative stress and inhibiting inflammatory mediators. They do this by either blocking KEAP1 or activating NRF2 expression, which then triggers the activation of its downstream targets in the nucleus. This process helps protect cells from damage and inflammation, contributing to disease prevention [73]. This makes it a compelling target for therapeutic strategies aimed at modulating oxidative stress and DNA repair mechanisms in both cancer and neurodegenerative diseases. Under normal conditions, NRF2 acts like a guardian, helping cells resist stress, repair damage, and avoid the first steps in cancer development. The problem arises when NRF2 is overactive in cancer cells, which can then exploit its protective mechanisms to resist therapy and survive [74]. In the context of cancer biology, the NRF2-KEAP1 pathway has a dual role. While NRF2 is essential for protecting normal cells from genotoxic stress, its sustained activation in cancer provides a survival advantage to tumor cells, contributing to drug resistance, metastasis, and poor prognosis [75]. Aberrant activation of NRF2, often caused by mutations in either KEAP1 or NRF2 itself, disrupts its normal degradation, leading to persistent nuclear accumulation and continuous activation of cytoprotective gene expression [76] (Fig. 3).

This sustained NRF2 activity enhances cancer cell survival by enabling adaptation to the oxidative and metabolic stress typical of the tumor microenvironment. As a result, NRF2-driven signaling promotes tumor progression and contributes to resistance against conventional therapies, representing a major challenge in cancer treatment [78,79]. NRF2 was originally recognized as a protein with cancer-preventive action, as it induces phase II enzymes and endogenous antioxidants that inactivate reactive carcinogens. Phase II detoxifying enzymes, such as glutathione S-transferase (GST) [80] and glucuronosyltransferase (UGT) [81], along with thiol-containing molecules and their generating enzymes, including glutamate cysteine ligase (GCL) and glutathione reductase (GR) [82,83], are responsible for metabolic detoxification involved in conjugation reactions in the liver. These conjugation reactions facilitate the excretion of reactive intermediates and/or carcinogens, protecting cells from redox cycling and oxidative stress. Stress response proteins such as heme oxygenase-1 (HO-1) [84] and direct ROS-scavenging enzymes like glutathione peroxidase (GPx) [85] can also be regulated by antioxidant response elements.

**Figure 4:**
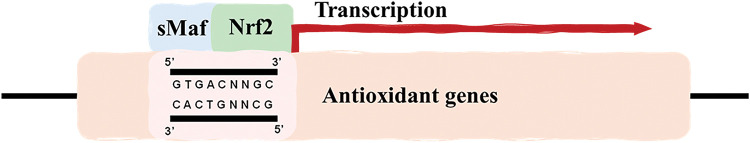
Core sequence of the ARE required for transcriptional activation of antioxidant genes. NRF2 heterodimerizes with sMaf proteins and binds to the ARE found within the promoter regions of antioxidant genes to activate their transcription. Adapted from [89].

The NRF2-KEAP1 signaling pathway is a critical cellular defense mechanism against oxidative and electrophilic stress. Under normal, unstressed conditions, KEAP1 acts as an adaptor protein for the CUL3-based E3 ubiquitin ligase complex, which targets NRF2 for ubiquitination and subsequent proteasomal degradation, thereby maintaining low levels of NRF2 in the cell [86,87]. Upon exposure to oxidative stress or electrophilic agents, specific cysteine residues on KEAP1 are modified, which disrupts its ability to target NRF2 for degradation. As a result, NRF2 accumulates in the cytoplasm, translocates to the nucleus, and binds to ARE in the DNA. This binding initiates the transcription of a variety of cytoprotective genes, including those involved in antioxidant defense, detoxification, and maintenance of redox homeostasis [87]. ARE is a regulatory element located in the enhancer region of many target genes that encode cytoprotective proteins [88]. It contains a specific DNA sequence—5′ GTGACNNGC 3′—with its complementary strand being—3′ CACTGNNCG 5′—**(Fig. 4)**, where “N” represents a variable nucleotide position [89].

**Figure 5:**
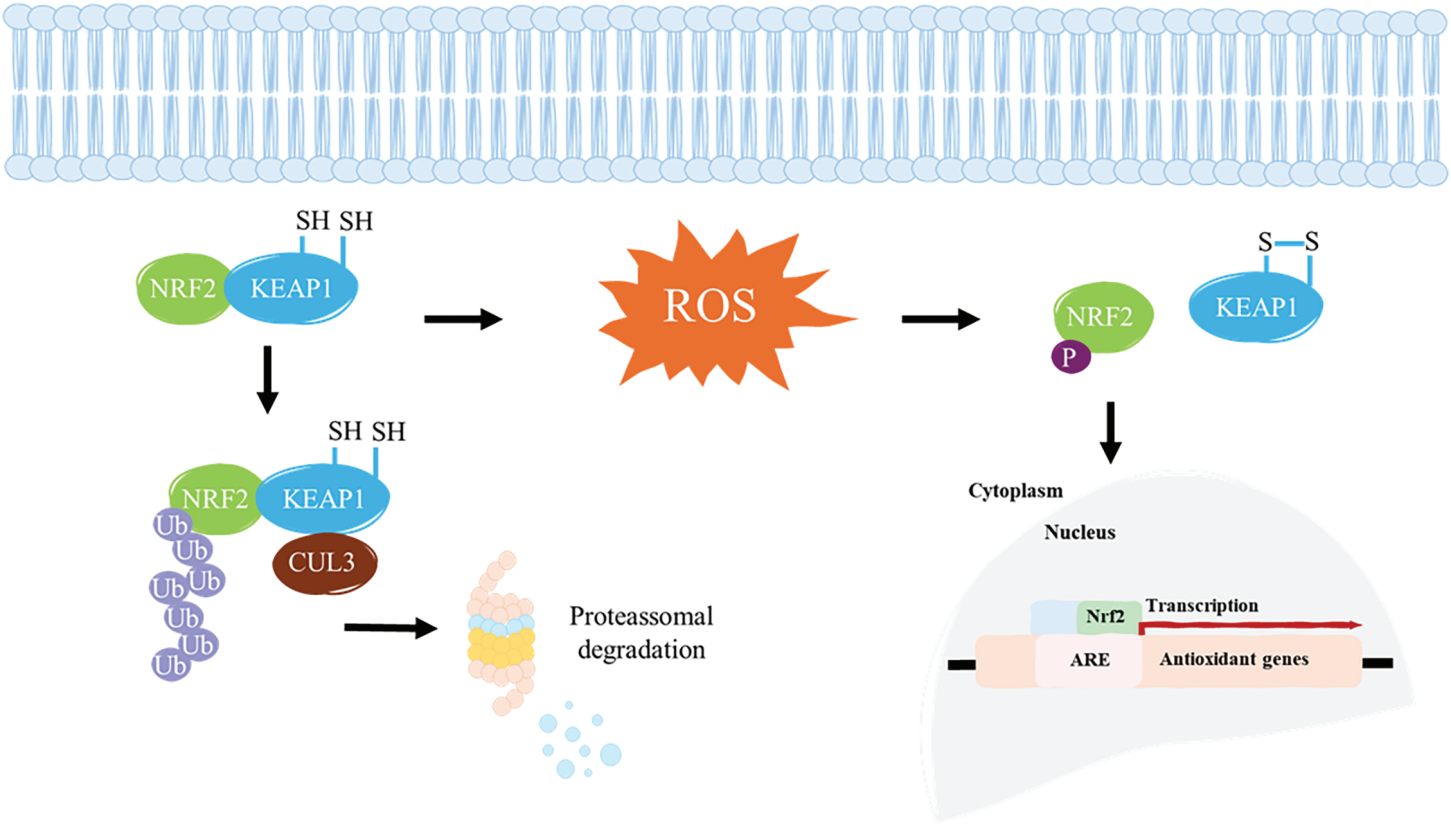
KEAP1 binds to NRF2, targeting it for proteasomal degradation under normal conditions. However, ROS induces conformational changes in KEAP1, disrupting its interaction with NRF2 and allowing it to escape degradation. Once freed, NRF2 translocates into the nucleus, where it binds to an ARE and interacts with key regulators to initiate the transcription of antioxidant genes. S–S, disulfide bond. Adapted from [111].

Studies have demonstrated a correlation between ARE and the basal gene expression levels of certain cytoprotective proteins, such as Glutathione S-transferase A2 (GSTA2) and Nicotinamide Adenine Dinucleotide Phosphate: Quinone Oxidoreductase 1 (NQO1) [90,91]. This modulation maintains homeostasis in response to oxidative stress generated by essential metabolic processes, such as mitochondrial respiration. ARE is present in the promoter region of genes encoding key detoxification enzymes, including GSTA2 and NQO1 [89]. GSTA2, primarily found in the liver, facilitates the neutralization of reactive species that may arise during the metabolism of certain molecules. NQO1 plays a crucial role in neutralizing ROS and compounds containing sulfhydryl groups within the intracellular environment [92]. These elements are recognized by nuclear transcription factors, primarily NRF2, which, upon activation, translocates to the nucleus and binds to AREs, promoting the transcription of genes involved in antioxidant defense and detoxification processes [91,93]. This mechanism is a key component of the cellular response to oxidative stress, contributing to the maintenance of redox homeostasis and protection against cellular damage. Under normal conditions, KEAP1 is responsible for maintaining low levels of NRF2. However, continuous exposure to oxidative stress or electrophilic insults, modifications of cysteine residues on KEAP1 lead to the stabilization and nuclear translocation of NRF2. Once in the nucleus, NRF2 binds to AREs in the promoter regions of target genes, initiating the transcription of a wide array of antioxidant and detoxification enzymes, such as glutathione S-transferases (GSTs), NAD(P)H NQO1, and HO-1 [94]. Beyond its antioxidant role, NRF2 also modulates inflammatory pathways, suppressing pro-inflammatory cytokines and inhibiting NF-κB signaling, and supports mitochondrial homeostasis by regulating biogenesis, dynamics, and mitophagy [52,95]. Dysregulation of this pathway is linked to several diseases. In cancer, persistent NRF2 activation can promote tumor growth and therapy resistance by enhancing detoxification and drug efflux mechanisms. Conversely, reduced NRF2 activity in conditions like neurodegenerative and metabolic diseases can worsen oxidative damage and inflammation [96]. KEAP1 has dual functions in this pathway: besides detecting the redox state through cysteine residues, it can also alter the ubiquitination level of NRF2 according to the redox state [97]. The primary role of the transcription factor NRF2 is to act as the key executor of this pathway, inducing gene expression by binding to its regulatory element. While ARE determines the specific effects of signaling activation, only genes with an ARE in their promoter region are involved in NRF2-KEAP1-ARE signaling [87]. Targeting this pathway may represent a promising therapeutic strategy for the prevention and treatment of these diseases. For example, NRF2 activation has been shown to protect against neurodegenerative disorders, such as Alzheimer’s and Parkinson’s diseases [98], as well as cardiovascular diseases [99] and cancer [100]. Targeting components of the NRF2–KEAP1–ARE axis thus presents a promising strategy to sensitize resistant tumors, optimize therapeutic responses, and minimize adverse effects. Understanding these mechanisms is essential for the development of combination therapies and precision medicine approaches in oncology, given that modulation of the NRF2-KEAP1-ARE pathway plays a crucial function in cell protection and survival by regulating the antioxidant defense system, oxidative stress, inflammation, and disease progression **(Fig. 5)**.

Some evidence correlates this signaling pathway with metabolic reprogramming in cancer cells through different mechanisms [101–103]. Among them, the regulation of redox homeostasis, mitochondrial physiology, autophagy, proteostasis, immune system function, and metabolism is noteworthy [87]. This pathway can be mediated through interactions with other transcription factors, activators, repressors, and crosstalk with other signaling pathways. The activation of the NRF2-KEAP1-ARE pathway in cancer cells generally enhances cell survival and resistance to apoptosis, rather than promoting apoptosis. This pathway plays a crucial role in cellular defense against oxidative stress by enhancing the expression of antioxidant and cytoprotective genes. In cancer cells, NRF2 activation often leads to increased expression of anti-apoptotic proteins such as B-cell lymphoma 2 (Bcl-2) and Bcl-2-like protein 1 (Bcl-xL), which contributes to enhanced cell survival and drug resistance [104,105]. Specifically, NRF2 has been shown to upregulate Bcl-2 and Bcl-xL, which are key anti-apoptotic proteins, thereby reducing apoptosis and promoting cell survival in the context of oxidative stress and chemotherapeutic challenges [106]. This suggests that rather than enhancing the apoptotic response, the activation of the NRF2-KEAP1-ARE pathway in cancer cells typically supports mechanisms that prevent apoptosis. However, the NRF2-ARE-Keap1 signaling pathway plays a dual and somewhat paradoxical role in cancer biology, particularly in the context of anticancer drug resistance. Many cancer types show constitutive activation of NRF2, often due to mutations in KEAP1 or NRF2 itself [107], this persistent activation leads to the upregulation of cytoprotective genes [108], which can neutralize chemotherapy-induced ROS [109], enhance drug efflux via transporters like multidrug resistance-associated protein 1 (MRP1/ABCC1) [110], maintain redox homeostasis even under chemotherapeutic pressure [86].

Clinical studies have shown that targeting the NRF2-KEAP1-ARE pathway can help overcome cisplatin resistance [66]. In head and neck squamous cell carcinoma (HNSCC), abnormal activation of the NRF2 pathway, often due to KEAP1 mutations or epigenetic alterations, has been closely associated with resistance to cisplatin and increased metastasis [112]. In urothelial carcinoma, prolonged cisplatin exposure leads to sustained NRF2 activation, which contributes to the development of drug resistance. Knocking down NRF2 in cisplatin-resistant urothelial carcinoma cell lines reduced the expression of cytoprotective enzymes and restored sensitivity to the drug [113]. While results may vary depending on the cell line, these findings support the idea that NRF2 inhibition could enhance the effectiveness of chemotherapy in certain cases. The NRF2-KEAP1-ARE pathway represents both a protective mechanism and a potential driver of therapeutic resistance in cancer. In early-stage cancer or prevention, NRF2 activation may be beneficial and in advanced or treatment-resistant cancers, targeted inhibition of NRF2 may enhance drug efficacy. Future research must focus on deciphering tumor-specific regulation of this pathway, developing selective modulators of NRF2, and understanding crosstalk with other signaling pathways.

p53, known as the “guardian of the genome,” plays a crucial role in regulating cell cycle arrest and apoptosis in response to DNA damage and oxidative stress [114]. It has a complex relationship with the NRF2 pathway [115]. Under low levels of ROS, p53 can enhance NRF2 activity to promote cell survival. However, at high levels of ROS, p53 suppresses NRF2 to facilitate apoptosis, thus balancing cell survival and death [116]. This biphasic regulation by p53 is essential for its tumor-suppressor function, coordinating the cellular response to stress and damage. Furthermore, mutant forms of p53 can interact with NRF2, altering its activity and supporting cancer cell survival. Mutant p53 can enhance the nuclear presence of NRF2 and modulate the transcription of specific NRF2 target genes, which can contribute to poor prognosis in certain cancers [117]. Overall, the interplay between the NRF2-KEAP1-ARE pathway, Bcl-2/Bcl-xL upregulation, and p53 protein underscores a complex network of interactions that regulate cell survival, apoptosis, and cancer progression. These pathways are interconnected through their roles in managing oxidative stress and cellular damage, with significant implications for cancer biology and therapy.

## Drug Resistance in Cancer Treatment

The emergence of drug resistance in cancer cells, where cells develop mechanisms to evade the effects of anticancer drugs, poses a significant challenge in cancer treatment, reducing the efficacy of many therapies. One key driver of drug resistance is oxidative stress, which activates survival pathways that allow cancer cells to persist and proliferate despite drug exposure. By modulating oxidative stress and interacting with critical signaling pathways, cancer cells can adapt to chemotherapy and targeted therapies, making them more resistant to treatment and ultimately diminishing therapeutic effectiveness [118]. NRF2, a transcription factor central to the antioxidant defense system, is essential for protecting cells from oxidative stress. However, its aberrant activation in cancer can contribute to tumor progression and therapy resistance by upregulating antioxidant defenses, detoxification enzymes, and drug efflux transporters [119]. This mechanism affects the efficacy of several anticancer drugs, including cisplatin, 5-fluorouracil, gemcitabine, oxaliplatin, paclitaxel, and doxorubicin, leading to reduced treatment effectiveness [120]. NRF2 activation leads to increased expression of phase I and phase II drug-metabolizing enzymes and phase III transporters, which can metabolize and extrude these drugs, reducing their efficacy [121,122]. The literature also highlights the role of NRF2 in regulating autophagy and the unfolded protein response (UPR), which are mechanisms that cancer cells exploit to survive under therapeutic stress, thereby contributing to chemoresistance [123]. Moreover, the NRF2-KEAP1 interaction is crucial in maintaining redox balance, and its dysregulation is associated with poor prognosis and selective drug resistance across various cancer types [124]. Efforts to overcome this resistance include targeting the NRF2 pathway with inhibitors, particularly those derived from plant sources, which have shown potential in reducing multidrug resistance [125]. However, the development of specific and non-toxic NRF2 inhibitors remains a challenge [71].

Platinum-based chemotherapies, such as cisplatin, carboplatin, and oxaliplatin, are widely used in cancer treatment due to their ability to induce oxidative stress and trigger cancer cell death. These agents primarily function by forming DNA adducts, leading to DNA damage and apoptosis. However, the involvement of the NRF2-p21-p53 pathway in drug resistance complicates their efficacy. When activated, the NRF2 pathway enhances the expression of antioxidant enzymes such as glutathione peroxidase and catalase, which mitigate ROS levels and protect cancer cells from oxidative damage, thereby contributing to anti-cancer drug resistance [126]. Similarly, topoisomerase inhibitors, such as doxorubicin and etoposide, and taxanes, such as paclitaxel and docetaxel, play crucial roles in cancer treatment by targeting different cellular mechanisms. However, their efficacy is often compromised by drug resistance, which can be influenced by oxidative stress and the NRF2-p21-p53 pathway [127,128]. Activation of NRF2 can enhance the expression of antioxidant enzymes, reducing ROS levels and protecting cancer cells from oxidative damage and influencing drug metabolism and efflux, thereby contributing to resistance [129].

Targeted therapies, including tyrosine kinase inhibitors (TKIs) such as erlotinib and sorafenib, and monoclonal antibodies, have distinct mechanisms of action compared to traditional chemotherapies like topoisomerase inhibitors and taxanes [130]. These targeted agents often exploit specific molecular pathways in cancer cells, but they are not immune to the challenges of drug resistance, which can be influenced by oxidative stress and the NRF2-p21-p53 pathway. Erlotinib and sorafenib, as TKIs, target specific kinases involved in cancer cell proliferation and survival. The role of oxidative stress in the action and resistance of these agents is increasingly recognized [131]. For instance, the NRF2 pathway, a key regulator of the antioxidant response, can be upregulated in response to oxidative stress, leading to enhanced survival of cancer cells and resistance to TKIs [132]. In hepatocellular carcinoma (HCC) and acute myeloid leukemia (AML), NRF2 activation has been associated with increased resistance to chemotherapy. This is due to its role in upregulating genes that enhance the cell’s ability to detoxify chemotherapeutic agents and scavenge ROS, thereby reducing drug efficacy [133,134]. In AML, pharmacological inhibition of NRF2 has been shown to improve sensitivity to chemotherapeutic agents by decreasing the expression of its downstream antioxidant targets, suggesting that NRF2 could be a potential target for overcoming drug resistance [134]. NRF2 is a central player in cellular defense mechanisms, modulating resistance to oxidative stress, inflammation, and chemotherapeutic agents through its regulation of antioxidant and detoxification pathways. Its dual role in protecting normal cells and contributing to cancer cell survival and chemoresistance highlights the complexity of targeting NRF2 in therapeutic strategies. This is supported by evidence that NRF2 activation can contribute to metabolic changes and drug resistance in cancer, as it regulates the expression of genes involved in antioxidant defense and drug metabolism [135]. Monoclonal antibodies, which target specific antigens on cancer cells, can also be affected by oxidative stress-related mechanisms. The modulation of oxidative stress pathways, including those regulated by NRF2, can influence the efficacy of these therapies. The NRF2 pathway’s role in maintaining redox homeostasis and its potential to support tumor survival under therapeutic pressure highlights its involvement in resistance mechanisms [136]. Targeting the NRF2 pathway, either directly or through its regulatory mechanisms, may offer new strategies to overcome resistance to targeted therapies, including TKIs and monoclonal antibodies [132]. This approach could enhance the efficacy of these treatments by disrupting the protective antioxidant responses that cancer cells exploit to survive therapeutic interventions.

There’s increasing interest in developing NRF2 inhibitors to combat the anti-drug resistance that often results from its overactivation in cancer cells [137]. However, one of the major challenges is designing compounds that are both effective and selective; many of the current options lack specificity and can be highly toxic. One promising approach involves creating small molecules that promote degradation of NRF2 by enhancing its interaction with KEAP1 or beta-transducin repeat–containing protein (β-TrCP), an E3 ubiquitin ligase adaptor protein. Enhancing β-TrCP activity or mimicking its binding could help reduce aberrant NRF2 accumulation and thereby counteract chemoresistance [138]. Natural compounds derived from plants have also been investigated for their ability to modulate NRF2 activity. These may offer a safer, less toxic alternative to synthetic inhibitors and have shown potential in overcoming multidrug resistance in cancer models [139]. Additionally, recent advances in medicinal chemistry have led to the development of small molecules that target the interaction between KEAP1 and NRF2. By disrupting this interaction, these inhibitors aim to regulate NRF2 levels and shift the redox balance within cancer cells [140]. For example, MSU38225 is a small molecule that has been shown to inhibit the NRF2 pathway and sensitize cancer cells to chemotherapy. It works by enhancing the ubiquitination and degradation of NRF2, thereby lowering the expression of NRF2 target genes and making cancer cells more responsive to treatment [141]. Overall, while our understanding of the NRF2-KEAP1-ARE pathway has significantly advanced, translating these insights into clinical therapies remains a challenge. Issues such as drug specificity, toxicity, and identifying which patients are most likely to benefit still need to be addressed through further research.

## The Complex Crosstalk between NRF2 and p53 in Oxidative Stress and Cancer

The complex interplay between the NRF2-KEAP1-ARE pathway, p53, and Bcl-2 family proteins has profound implications for cancer therapy, particularly in treatment resistance and targeted interventions. In many cancers, NRF2 is constitutively activated due to mutations in KEAP1, overexpression of NRF2, or aberrant oncogenic signaling, leading to enhanced antioxidant defenses [104]. This persistent activation protects cancer cells from ROS-induced apoptosis and contributes to increased resistance to chemotherapy and radiotherapy, as these treatments often rely on oxidative damage to eliminate cancer cells [116]. Additionally, NRF2-driven metabolic reprogramming supports tumor progression by promoting glycolysis, glutamine metabolism, and fatty acid synthesis, ensuring a continuous supply of energy and biosynthetic precursors for rapid proliferation [142]. While NRF2 plays a crucial protective role in normal cells, its dysregulation in cancer fosters tumor survival and therapy resistance, making it a potential target for anticancer strategies.

The relationship between p53 and NRF2 depends on the level of cellular stress. Under low oxidative stress, p53 enhances NRF2 activation to promote antioxidant defenses and cell survival and under severe oxidative stress, p53 inhibits NRF2, promoting ROS accumulation and triggering apoptosis to eliminate damaged cells. This biphasic regulation ensures that cells survive mild stress but do not accumulate mutations under severe oxidative damage. Many cancers harbor mutant p53, which alters its interaction with NRF2, mutant p53 stabilizes NRF2, increasing the expression of antioxidant and detoxification genes [143]. This supports cancer cell survival and proliferation, contributing to anticancer drug resistance and poor prognosis. Some studies suggest that mutant p53 hijacks NRF2 to selectively activate pro-tumorigenic genes while suppressing tumor-suppressive pathways [117,144]. The mutant p53-NRF2 axis represents a pivotal mechanism through which cancer cells evade apoptosis, enhance their survival, and develop resistance to therapeutic interventions. This interplay not only sustains high antioxidant capacity but also rewires cellular metabolism to support tumor progression, making it a promising target for innovative cancer therapies. Furthermore, the NRF2-KEAP1-ARE pathway, p53, and Bcl-2 family proteins form a highly interconnected regulatory network that governs cancer cell fate. While NRF2 plays a fundamental role in maintaining redox homeostasis and cytoprotection, its persistent activation in malignancies, especially in p53-mutant tumors, drives tumor survival, therapy resistance, and metabolic reprogramming. Targeting this intricate crosstalk holds significant therapeutic potential, offering opportunities to selectively inhibit NRF2 in cancer cells while preserving its essential protective functions in normal tissues, thus minimizing collateral damage and improving treatment efficacy.

Among the molecular mechanisms involved, the NRF2-p21-p53 signaling pathway plays a crucial role in regulating the cellular response to oxidative damage and anticancer therapies. The interaction between oxidative stress, this pathway, and drug resistance is complex and multifaceted [145]. In cancer cells, mutations in p53, hyperactivation of NRF2, and overexpression of p21 (CDKN1A) disrupt normal cell cycle regulation, enhance cell survival, and drive therapy resistance. This combination allows cancer cells to evade apoptosis, persist under oxidative stress, and resist chemotherapy and radiation [146]. p21 is known to directly interact with NRF2, enhancing its stability and thereby promoting the NRF2-mediated antioxidant response. This interaction occurs through p21 competing with KEAP1 for binding to NRF2, which reduces NRF2 ubiquitination and degradation, thus facilitating its activation [147]. This mechanism underscores the importance of p21 in modulating the cellular response to oxidative stress via NRF2. As a cyclin-dependent kinase (CDK) inhibitor, p21 primarily functions downstream of p53 to induce cell cycle arrest, facilitating DNA repair and maintaining genomic stability. However, p21 also exhibits anti-apoptotic properties, which can counteract p53-driven cell death under certain conditions [148]. It has been observed that p53 can modulate NRF2 expression in a biphasic manner: at low levels of ROS, p53 enhances NRF2 expression to promote cell survival, while at high ROS levels, p53 suppresses NRF2 to facilitate apoptosis. This regulation is p21-dependent, highlighting p21’s role as a mediator in the p53-NRF2 interaction [149]. Additionally, the modulation of NRF2 activation by p53 is influenced by long noncoding RNAs (lncRNAs), which function as miRNA sponges for p21 and other regulatory proteins, fine-tuning the p53-NRF2 axis. This mechanism reinforces the role of p21 in the p53-dependent activation of NRF2 under stress conditions, such as DNA damage and serum deprivation [150]. Moreover, p21 stabilizes NRF2 by inhibiting Glycogen Synthase Kinase 3 Beta (GSK-3β), a serine/threonine kinase, preventing its degradation and enhancing the antioxidant response, thereby promoting cell survival [151]. In cancers with dysregulated p53 or hyperactive NRF2 signaling, p21’s ability to promote cell survival over apoptosis can contribute to anticancer drug resistance and tumor progression. Thus, understanding the complex interplay between p21, NRF2, and p53 is crucial for developing therapeutic strategies that can modulate oxidative stress responses, enhance treatment efficacy, and restore tumor-suppressive mechanisms, ultimately improving cancer treatment outcomes. The NRF2-p21-p53 signaling pathway plays a critical role in cancer drug resistance by enhancing cell survival mechanisms and promoting drug efflux, ultimately reducing the efficacy of anticancer therapies. This pathway regulates the cellular response to DNA damage and oxidative stress, which are key mechanisms through which many chemotherapeutic agents exert their cytotoxic effects. p53, a tumor suppressor, is involved in DNA repair and apoptosis; however, in the context of chemoresistance, p53 activity can be altered to favor cell survival instead of apoptosis, enabling cancer cells to withstand treatment. The pathway can induce the production of antioxidants and multidrug resistance-associated proteins, which protect cancer cells from therapy-induced oxidative stress and promote drug efflux. Overall, the NRF2-p21-p53 pathway contributes to resistance by enhancing antioxidant defenses, promoting drug efflux, and modulating DNA repair processes, thereby supporting cancer cell survival and reducing the efficacy of anticancer drugs like topoisomerase inhibitors and taxanes [128]. Understanding these mechanisms offers potential targets for overcoming resistance and improving therapeutic outcomes.

## Conclusions

Oxidative stress plays a major role in how cancer cells develop resistance to anticancer drugs, largely through the NRF2-KEAP1-ARE signaling pathway. When cells experience stress, NRF2 becomes activated and triggers the production of antioxidant enzymes, detoxification proteins, and other protective factors that help cells survive. While this response is meant to protect healthy cells, in cancer, it can backfire. Mutations or disruptions in the NRF2-KEAP1 interaction can lead to constant NRF2 activation, creating a sustained antioxidant response that shields tumor cells from chemotherapy-induced damage. This not only helps cancer cells survive but also fuels tumor progression. Because of the crucial role this pathway plays in drug resistance, targeting NRF2-KEAP1-ARE could be a promising approach to restoring the balance between oxidative stress and cell survival. By doing so, we may be able to make cancer cells more responsive to chemotherapy and improve treatment outcomes. The key players in this pathway include NRF2 is a transcription factor encoded by the NFE2L2 gene, which plays a central role in regulating the expression of antioxidant proteins that protect against oxidative damage triggered by injury and inflammation. Under normal conditions, NRF2 is bound by KEAP1 in the cytoplasm, which targets it for ubiquitination and subsequent proteasomal degradation. KEAP1 is an E3 ubiquitin ligase adaptor protein that regulates NRF2 by promoting its degradation under homeostatic conditions [152]. KEAP1 contains cysteine residues that act as sensors for oxidative and electrophilic stress. When these cysteines are modified, KEAP1 undergoes a conformational change that prevents it from targeting NRF2 for degradation, allowing NRF2 to accumulate and translocate to the nucleus [58]. ARE—The Antioxidant Response Element is a specific DNA sequence found in the promoter region of various genes encoding antioxidant and cytoprotective proteins. Once NRF2 is released from KEAP1 and translocates to the nucleus, it binds to AREs, initiating the transcription of genes involved in the detoxification and elimination of reactive oxidants and electrophiles [153]. The NRF2-KEAP system is a thiol-based sensor-effector apparatus that maintains redox homeostasis. The rapid inducibility of NRF2 in response to stress is a hallmark of this pathway, allowing cells to swiftly counteract oxidative damage. This pathway is implicated in various diseases, including cancer, where dysregulation can lead to enhanced survival of cancer cells under oxidative stress conditions [154]. Understanding and targeting this pathway holds therapeutic potential for diseases characterized by oxidative stress and inflammation. To effectively harness the NRF2-KEAP1-ARE pathway for cancer therapy, several key research directions must be prioritized. One major focus is the context-dependent role of NRF2, which can act as either a tumor suppressor or promoter depending on the cellular and tumor microenvironment. Understanding the molecular mechanisms that dictate this duality is essential. Another critical area involves NRF2’s role in cancer metabolism. As NRF2 supports metabolic reprogramming in tumor cells—particularly under nutrient-limited conditions—mapping these NRF2-regulated pathways could uncover novel therapeutic vulnerabilities. Additionally, noncanonical mechanisms of NRF2 activation are gaining attention. Exploring these alternative regulatory routes may reveal new therapeutic targets or biomarkers relevant across different cancer types. Developing selective and safe NRF2 inhibitors remains a significant challenge due to the protein’s complex structure and its widespread physiological functions. Continued medicinal chemistry efforts are needed to design molecules that effectively modulate NRF2 activity, especially in tumors with constitutive NRF2 activation linked to drug resistance. Furthermore, large-scale genomic and transcriptomic studies could identify patient subgroups most likely to benefit from NRF2-targeted interventions, thereby supporting a more precise and personalized approach to treatment. The crosstalk between NRF2 and other signaling pathways, including those related to inflammation, microRNAs, and immune responses, also deserves closer investigation. These interactions may influence cancer progression and treatment outcomes in ways not yet fully understood. Despite encouraging preclinical data, NRF2-targeting agents have not yet achieved clinical impact. Carefully designed clinical trials are needed to evaluate optimal timing, dosage, and patient selection for these therapies, ideally in combination with existing cancer treatments. Ultimately, progress in this field will depend on a multidisciplinary approach that integrates molecular biology, pharmacology, systems biology, and clinical oncology. Such collaboration is essential to realize the full therapeutic potential of modulating the NRF2-KEAP1-ARE pathway in cancer care.

## Data Availability

Not applicable.
